# Quantitative evaluation of rat sciatic nerve degeneration using high-frequency ultrasound

**DOI:** 10.1038/s41598-023-47264-9

**Published:** 2023-11-18

**Authors:** Yuanshan Wu, Victor Barrere, Aiguo Han, Michael P. Andre, Elisabeth Orozco, Xin Cheng, Eric Y. Chang, Sameer B. Shah

**Affiliations:** 1 Department of Bioengineering, University of California, San Diego, 9500 Gilman Drive, MC 0863, La Jolla, CA 92093-0683 USA; 2 Department of Orthopaedic Surgery, University of California, San Diego, La Jolla, CA USA; 3 Research Service, VA San Diego Healthcare System, San Diego, CA USA; 4 Department of Biomedical Engineering and Mechanics, Virginia Polytechnic Institute and State University, Blacksburg, VA USA; 5 Department of Radiology, University of California, San Diego, La Jolla, CA USA

**Keywords:** Biomedical engineering, Peripheral nervous system, Imaging techniques

## Abstract

In this study, we evaluated the utility of using high-frequency ultrasound to non-invasively track the degenerative process in a rat model of peripheral nerve injury. Primary analyses explored spatial and temporal changes in quantitative backscatter coefficient (BSC) spectrum-based outcomes and B-mode textural outcomes, using gray level co-occurrence matrices (GLCMs), during the progressive transition from acute to chronic injury. As secondary analyses, correlations among GLCM and BSC spectrum-based parameters were evaluated, and immunohistochemistry were used to suggest a structural basis for ultrasound outcomes. Both mean BSC spectrum-based and mean GLCM-based measures exhibited significant spatial differences across presurgical and 1-month/2-month time points, distal stumps enclosed proximity to the injury site being particularly affected. The two sets of parameters sensitively detected peripheral nerve degeneration at 1-month and 2-month post-injury, with area under the receiver operating charactersitic curve > 0.8 for most parameters. The results also indicated that the many BSC spectrum-based and GLCM-based parameters significantly correlate with each other, and suggested a common structural basis for a diverse set of quantitative ultrasound parameters. The findings of this study suggest that BSC spectrum-based and GLCM-based analysis are promising non-invasive techniques for diagnosing peripheral nerve degeneration.

## Introduction

Peripheral nerve injury severely impairs an individual’s sensorimotor function^1^, and often results in chronic muscular disability and pain^2^. The severity of injured nerve tissue and its potential for regeneration depends significantly on the timing of repair after the injury^3^. When unrepaired, nerves progressively undergo a number of structural and biological changes, including increases in the expression and persistence of inflammatory markers, fibrosis, and reduced pro-regenerative signaling from Schwann cells in the distal stump^3–5^. Though nerves repaired beyond two to three months are typically considered chronically injured^6^, the progressive nature of degeneration results in substantial variability in the regenerative environment within and across species^7–9^. Clinically, nerve injury is typically diagnosed using electrophysiological testing, which effectively reveals impaired nerve conduction^10, 11^. However, such testing does not speak to the underlying cause of damage or assess the quality of nerve tissue. Consequently, there is high value in developing novel diagnostic tools that accurately assess the degenerative or regenerative environment.

Medical imaging allows qualitative assessment of nerve continuity and impingement of the nerve by surrounding tissues^12, 13^. Among the medical imaging modalities, ultrasound (US) is a widely used, affordable, and effective diagnostic modality for nerve imaging^14^. High-frequency US scanners (often defined as > 15 MHz for clinical application) have been recently made available for clinical use and provide remarkably high-resolution assessments of tissue structure and pathology^15–17^. For example, evaluation of B-mode images from high-frequency US systems complements the clinical evaluation of peripheral neuropathies such as carpal tunnel syndrome as well as injury^18–20^. Beyond enabling improved subjective B-mode assessment, high-frequency US imaging additionally facilitates high-resolution assessment of tissue speckle characteristics, including extraction of image texture features via gray level co-occurrence matrices (GLCMs)^21^. Such approaches have been used in the textural analysis of nerves in patients diagnosed with amyotrophic lateral sclerosis^22^. High-frequency quantitative ultrasound (QUS) has also been deployed for the characterization of attenuation-based and backscatter coefficient (BSC) spectrum-based outcomes in tissues. QUS exploits information in raw beam-formed radiofrequency (RF) data, and upon system calibration using reference phantoms, enables the system-independent measurement of fundamental acoustic properties of a given target^23, 24^. This approach has yielded microstructure-pathology relationships in various tissues such as the liver, kidney, red blood cells, prostate, and breast^25–29^.

The deployment of high-frequency QUS approaches to evaluate peripheral nerve remains limited. A recent study demonstrated the feasibility of high-frequency QUS and GLCM-based approaches for evaluating human median nerves *in vivo*^30^, building upon similar assessments in cadaveric nerves, which demonstrated correlation of QUS-based and GLCM-based outcomes with levels of collagen and myelin^31^. However, these approaches have not yet been deployed in a diagnostic context, nor have they been used in animal models of nerve injury or disease. In this study, we evaluated the utility of using high-frequency BSC spectrum-based and GLCM-based outcomes to non-invasively track the degenerative process in a rat model of peripheral nerve injury. Imaging outcomes were interpreted in the context of progressive morphological changes occurring during the transition from acute to chronic injury.

## Materials and methods

### Animal model

All methods have been carried out in accordance with relevant guidelines and regulations. The study was designed and carried out in compliance with the ARRIVE guidelines on animal research^32^. Approval for this study was obtained from the Veterans Affairs San Diego Healthcare System Institutional Animal Care and Use Committee (IACUC). Thirteen 300 g male Lewis rats (6–8 weeks old) were obtained from Charles River Laboratory Inc, Wilmington, MA. All animals underwent survival surgery under isoflurane anesthesia (3%) with preoperative subcutaneous injection of 1.0 mg/kg sustained release buprenorphine for analgesia, 50 mg/kg of cefazolin as an antibiotic, and 2mL/kg of 0.9% NaCl for hydration. Animals were euthanized by carbon dioxide inhalation.

**Figure 1:**
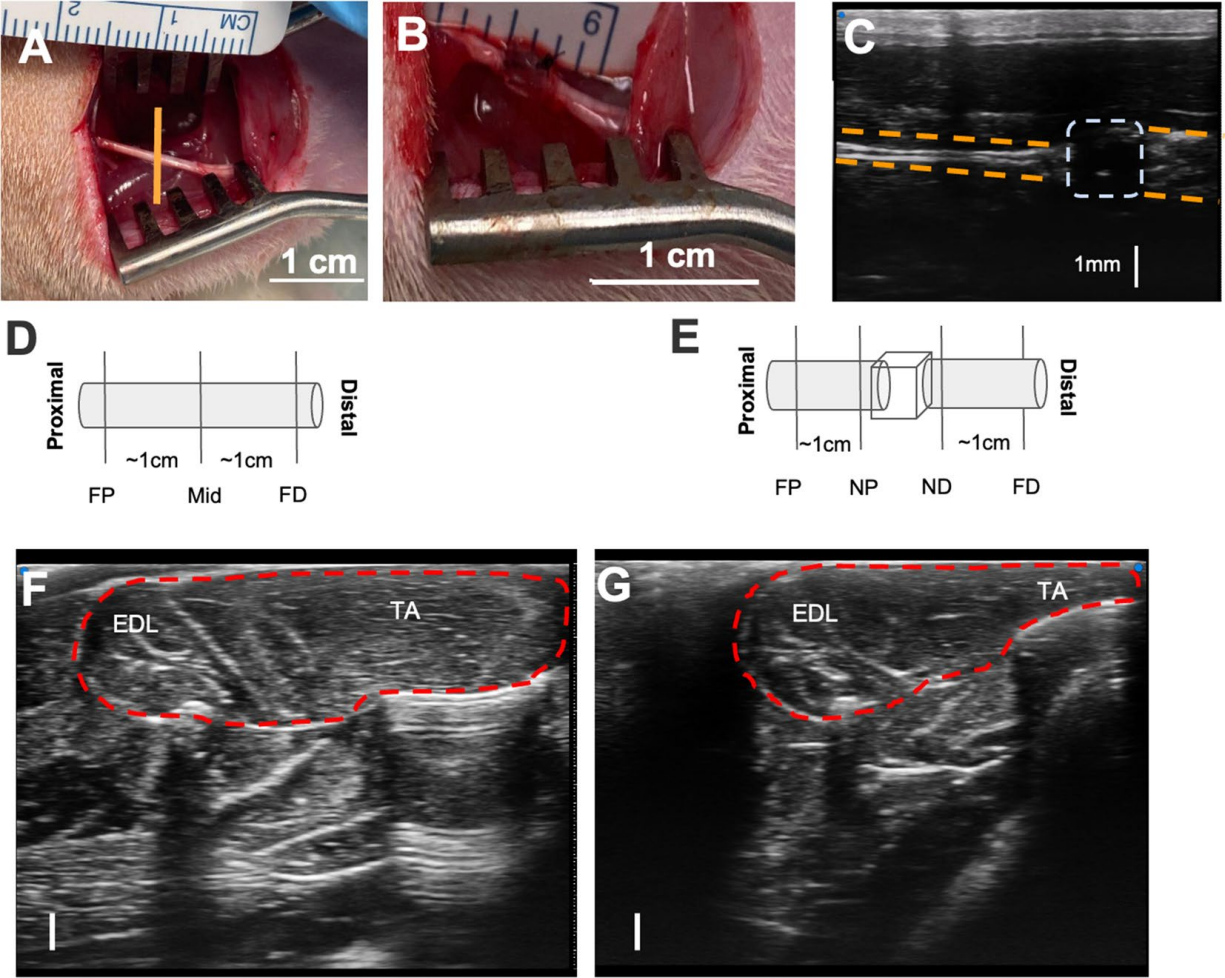
Sciatic nerve exposure, transection, and polydimethylsiloxane implantation. **(A)** 2 cm longitudinal incision was made 1 cm posterior to the femur (the location of orange vertical line). The sciatic nerve was exposed by dissecting through the biceps femoris muscle. **(B)** The transection was made 1 cm proximal to the trifurcation and a PDMS implant was inserted to both prevent regrowth and serve as an imaging landmark. **(C)** Longitudinal B-mode image showing the sciatic nerve (orange dashed lines) and PDMS implant (gray dashed box). **(D)** Schematic of the intact, pre-surgical sciatic nerve at the location of the ultrasound acquisition. The far proximal (FP), middle (Mid), and far distal (FD) locations are labeled. **(E)** Schematic of the post-surgical nerve, with additional near proximal (NP) and near distal (ND) locations labeled. **(F)** B-mode image of the tibialis anterior (TA) and extensor digitorum longus (EDL) muscles before surgery. **(G)** B-mode image obtained 2 months post-injury. The red dot line is the border of the muscle group. The scale bar represents 1 mm in the axial direction for both **(F,** **G)**.

Right sciatic nerves were exposed at mid-thigh level via a single 2–3 cm long incision through the skin and overlying tissue. Nerves were decompressed from their bed and sharply transected 1 cm proximal to their trifurcation (Fig. 1A). Nerves were capped with an autoclaved polydimethylsiloxane (PDMS) block to prevent spontaneous regeneration, thereby creating a model of progressive nerve degeneration^33^. The block also served as an imaging landmark (Fig. 1B). Fascia was closed with 4–0 monofilament nylon sutures and the skin was closed with staples (Stoelting™ EZ Clip, Stoelting, IL USA).

### Ultrasound

US imaging (Vevo MD, Fujifilm, Toronto, Ontario, Canada) was performed at three different time points (pre-op, 1 month post-surgery, and 2 months post-surgery), corresponding to the degenerative transition from acute to chronic nerve injury within each animal^8, 34^. Images were acquired from the right hind limbs of rats under 2% isoflurane anesthesia with a high-frequency probe (UHF 48 with bandwidths of 11–30 MHz). Three raw beamformed RF frames of data were acquired using the scanner’s research mode. Three locations, far proximal (FP), far distal (FD), and middle (Mid), were scanned pre-surgically (Fig. 1D) while four locations, far proximal (FP), near proximal (NP), far distal (FD), and near distal (ND), were scanned post-surgically (Fig. 1E); the pre-operative Mid location was compared to both NP and ND locations post-transection. US settings were consistent across all acquisitions with imaging depth of 11.5 mm and focal zone at 4.7 mm. Dynamic range was set to 65 dB and the acquisition gain was set to 40 dB for all acquisitions. Reference RF data was acquired on QUS phantom RDG 5435 (~ 25 mm thickness, ~ 30 mm radius; attenuation coefficient $\alpha_{ref} \left( f \right) = 0.2089f^{1.4183} + 0.2712$; the speed of sound 1544 m/s; BSC ~ -20 dB at 20 MHz; CIRS, Norfolk, VA, USA) with the same US settings^30^. All acquisition settings were selected by a trained musculoskeletal ultrasound scientist and confirmed by a board-certified radiologist to optimize the signal and image quality. Sample B mode images of tibialis anterior (TA) and extensor digitorum longus (EDL) muscles were also assessed by placing the transducer transversely at the muscle belly of TA at half of the distance on the line from patella to the lateral malleolus with general B mode presets with imging depth at 11.5 mm and focal zone at 4.7 mm. Dyanmic range was set to 65 dB, acquisition gain was 31 dB, and time gain compensation (TGC) was 46–61 dB (Fig. 1F, G).

### Immunohistochemistry

A subset of rats was sacrificed at 1-month post-injury (n = 4) and 2-month post-injury (n = 4) for immunohistochemical (IHC) analysis. IHC was performed with minor modifcations to a previously published protocol^35^. Briefly, proximal and distal stumps were preserved in 4% paraformaldehyde for 24 h at 8ºC and stored in 70% ethanol. Tissues were blocked into the four regions designated based on ultrasound acquisition locations (FD, ND, FP and NP; Fig. 1E). Standard paraffin embedding, deparaffinization, and antigen retrieval protocols were followed prior to obtaining 5 µm cross Sects.^35^. Primary antibodies used were rabbit polyclonal anti-laminin antibody (PA1-16730, Invitrogen, Waltham, MA, US) and mouse monoclonal anti-beta3-tubulin antibody (MA1-118, Invitrogen, Waltham, MA, US), each at 1:500 dilution. Secondary antibodies used were AlexaFluor488 goat-anti-rabbit at 1:300 dilution and AlexaFluor 568 goat-anti-mouse at 1:100 dilution. Epifluorescence imaging was conducted using a Leica DM6000 microscope with a 20X/0.70 objective.

Masson’s trichrome staining was also performed on the samples described above, using a commercially available kit (87019, Epredia, MI, US). Briefly, we first deparaffinized and hydrated the sections and placed them in Bouin’s fluid at 56 °C for 1 h. After rinsing, we stained sections in Working Weigert’s iron hematoxylin for 10 min and in Biebirch Scarlet-Acid Fuchsin Solution for another 5 min. Finally, we placed the sections in Phosphotungstic-Phosphomolybdic acid for minutes and stained with Aniline Blue for 5–10 min. After coverslipping, slides were scanned (Axio scanner 7, Zeiss, Germany) with 5 × and 10 × objectives. Images were segmented by color thresholding. Following background subtraction, red–green–blue (RGB) RGB images were transformed into luminance-bandwidth-chromiance (YUV) color space to more easily separate the blue stain (connective tissue). The ratio of blue area to the area of the entire section was calculated as the area fraction of connective tissue.

### Analytical methods

Briefly, BSC (${\text{cm}}^{ - 1} \;{\text{Sr}}^{ - 1}$) is defined as the differential backscatter cross-section per unit volume (i.e., an indicator of the relative power re-emitted from the medium towards the emitter for each frequency in the bandwidth). BSC was determined using the reference phantom method^36^ that explicitly accounts for experimental factors such as effects of focusing, time gain compensation and diffraction^37^ affecting the ultrasound signal.

1 $$BSC_{tissue} \left( f \right) = BSC_{ref} \left( f \right)\frac{{P_{tissue} \left( f \right)}}{{P_{ref} \left( f \right)}}10^{{4d\left[ {\alpha_{tissue} \left( f \right) - \alpha_{ref} \left( f \right)} \right] }}$$

where *f* is the frequency of the acoustic waves, $BSC_{ref} \left( f \right)$ is the BSC of the reference phantom material measured in a water bath with a single element transducer, $\alpha_{tissue} \left( f \right)$ is the attenuation of the tissue, $\alpha_{ref} \left( f \right)$ is the attenuation of the reference phantom (RDG 5435) noted above, $P_{tissue} \left( f \right)$ is the power spectrum from the tissue, $P_{ref} \left( f \right)$ is the power spectrum from the phantom (single frame) and *d* is the distance between the transducer and the region of interest (ROI). All BSCs reported in this study are logarithmically transformed, where $BSC = 10\log_{10} \left( {\frac{{BSC_{tissue} }}{{BSC_{0} }}} \right)$, with $BSC_{0} = 1 cm^{ - 1} .sr^{ - 1}$. We first calculated integrated BSC (iBSC) by integrating the BSCs between $f_{l}$ = 14 MHz and $f_{h}$ = 18 MHz, where $iBSC = \frac{1}{{f_{h} - f_{l} }}\mathop \smallint \limits_{{f_{l} }}^{{f_{h} }} BSCdf$^38^. Use of this frequency range allowed comparison with similar outcomes in a previous study^30^.In addition, we performed a similar analysis between $f_{l}$ = 18 MHz and $f_{h}$ = 26 MHz, spanning the central frequency of 22 MHz. Per published methods, linear regression analysis was performed on BSCs across the entire analyzed frequency range, to extract the slope and y-intercept (extrapolated spectral value to 0 MHz)^39^.

The attenuation *α(f)* (${\text{dB}}\;{\text{cm}}^{ - 1}$) is a fundamental property of tissue that indicates the loss of energy by absorption and scattering from the acoustic wave per unit distance of travel. The model for the frequency dependence of attenuation considers $\alpha = \beta f^{b}$, with β the power law prefactor in ${\text{dB}}\;{\text{cm}}^{ - 1} \;{\text{MHz}}^{ - b}$ and *b* is the unitless power law exponent for the frequency dependence of attenuation. Heterogeneity of attenuation of overlying muscle ($a_{Mus}$ = 0.54 ${\text{dB}}\;{\text{cm}}^{ - 1} {\text{MHz}}^{ - b}$; $b_{mus} = 1.33$), fat ($a_{fat}$ = 1.32 ${\text{dB}}\;{\text{cm}}^{ - 1} \;{\text{MHz}}^{ - b}$; $b_{fat} = 1.09$), and skin ($a_{skin}$ = 0.37 ${\text{dB}}\;{\text{cm}}^{ - 1} \;{\text{MHz}}^{ - b}$; $b_{skin} = 1.72$) was accounted for utilizing an approach in which the thickness of each layer and literature-based values^40–42^ for the attenuation of each tissue type are combined to calculate a composite attenuation for these layers; this approach bypasses analytical artifacts associated with tissue interfaces^30^.

### Image GLCM texture analysis

B-mode images reconstructed from RF data were utilized for textural analysis since these images were unaffected by the system settings. Then, the images were analyzed using the GLCM algorithm described by the following Eq. (2):

2 $$C_{{{\Delta }x,{\Delta }y}} \left( {i,j} \right) = \mathop \sum \limits_{x = 0}^{n - 1} \mathop \sum \limits_{y = 0}^{m - 1} \left\{ {\begin{array}{*{20}l} {1,} \hfill & { if\; I\left( {x,y} \right) = i \;and\; I\left( {x + \Delta x,y + \Delta y} \right) = j} \hfill \\ {0,} \hfill & {otherwise} \hfill \\ \end{array} } \right.$$

Briefly, the B-mode ultrasound image is treated as a matrix of grayscale pixel intensities, *I(x,y)* at the *x*th and *y*th location. (*i, j*) are the grayscale pixel intensity levels. 8 different gray levels were considered in this study. The offset Δ*x* is equal to 0 in the horizontal direction and Δ*y* is equal to *λ/*4 with *λ* the wavelength of the acoustic wave into the depth of the tissue. Contrast, homogeneity, correlation, energy and entropy features are measured as previously defined^21^ using MATLAB (v2020b, The Math Works, Natick, MA).

### Graphical user interface and ROI selection

The above outcomes were analyzed in MATLAB using a previously published estimator graphical user interface (GUI)^43^. Using the uncompressed and unfiltered reconstructed B-mode images, a trained musculoskeletal ultrasound scientist manually outlined the nerve ROIs, which were ovoid in shape and included the outer edge of the epineurium. The GUI automatically determined sub-ROIs within a given ROI; per guidance provided in prior studies, sub-ROI dimensions were selected to be > 10λ in the axial direction and > 10 A-lines in the lateral direction^44^. In this study, sub-ROIs of most images were > 12λ × 12 A-lines. Adjacent sub-ROIs overlapped by 75%. For the morpological assessments, the size of ROIs was computed by individal pixel sizes. Similarly, the ROIs of muscle B-mode images were also manually assessed by outlining the border of TA and EDL muscles (Fig. 1F, G) to compare the size of the muscle group.

### Statistics

The effect across time points and imaging sites were compared using two-way ANOVA**.** Within each individual imaging site, mean values were compared using one-way ANOVA with time point as the fixed effect. Significant differences were designated for *p < 0.05; **p < 0.01; ***p < 0.001. Post-hoc comparisons between individual experimental groups were performed by the Tukey’s range test, which accounts for multiple comparisons. Sample sizes for each group were (pre-op: n = 14; 1 month: n = 13; 2 months: n = 9). Minimum sample sizes (N = 7) were determined using G*Power 3.1.9.7^45, 46^ based on a power analysis with α = 0.05, conservative effect size = 0.25 (based on preliminary BSC spectrum-based outcomes), and power (1-β) = 0.8. No blinding or randomization was required due to the longitudinal study design. Standard Pearson correlation coefficients (r) were determined for combinations of BSC spectrum-based and GLCM-based parameters. The r values were interepreted according to the previous standard with 0.9–1.0, very high correlation; 0.7–0.9 high corrrelation; 0.5–0.7 moderate correlation; 0.3–0.5 low correlation; 0–0.3 negligible correlation^47^. IHC outcomes were correlated with US-based parameters by matching ultrasound images from a particular location and time with the corresponding immunolabeled or histological outcome from the same rat, and performing linear regression. The receiver operating charactersitic curve (ROC), which is defined as a plot of test sensitivity versus false positive rate, were also present. Area under the ROC curve (AUC) was reported in this study with AUC ≥ 0.9, excellent; 0.8 ≤ AUC < 0.9, good; 0.7 ≤ AUC < 0.8, fair; 0.6 ≤ AUC < 0.7, poor; AUC < 0.6, failed model discrimination^48^. Statistical analyses were performed using SPSS 28.0.1.0 (IBM Company, New York, USA).

## Results

**Figure 2:**
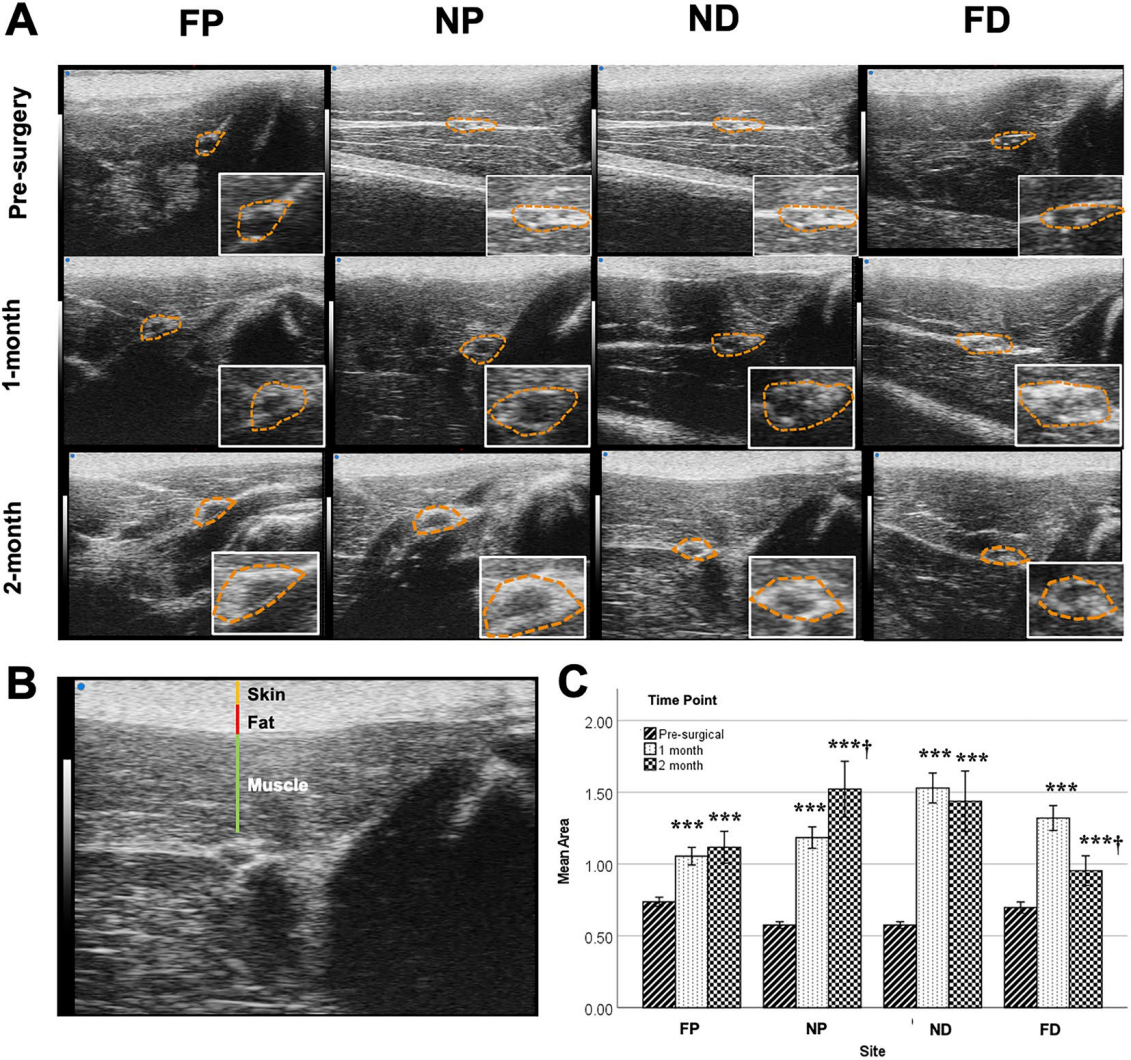
**(A)** Representative B-mode images of the sciatic nerve from a single rat at different time points (rows) and different imaging locations (columns). The nerve is enclosed inside an orange ROI from one of the rats. The scale bar is consistent throughout the images representing 1 mm in the axial direction. **(B)** One example B-mode image with different overlaying tissues identified. **(C)** Bar plot of the nerve area measurements at different time points and different sites. One-way ANOVA was conducted on the fixed factor time point with the Tukey HSD multiple comparison post hoc test. ***P < 0.001 versus pre-surgery time point, ^†^*P* < 0.001 versus 1-month time point. mean ± SEM.

We evaluated the execution of our transection nerve-capping injury model using B-mode US images of the nerve. Longitudinal images of each nerve confirmed the location of transection, the orientation of nerves, and the proper position of the PDMS cap (Fig. 1C), prior to cross-sectional image acquisitions used for analysis. TA and EDL muscle cross-sectional area was also compared between images acquired from pre-op and 2-month post-op animals. Substantial atrophy of both muscles was observed after injury, with a reduction in cross-sectional area of ~ 40% in representative images (Fig. 1F, G). Representative cross-sectional B-mode images of nerves are shown at each location at a given time point (Fig. 2A). To replicate typical morphological analyses performed on such images^49, 50^, cross-sectional areas of nerve ROIs in each image were measured; significant differences were observed between pre-surgery and post-surgery groups at all locations, although sites closest to the injury site (near proximal NP and near distal ND) were more substantially enlarged (~ 3x) compared to sites about 1 cm away from the injury (far proximal FP and far distal FD, ~ 1.5 × enlargement).

**Figure 3:**
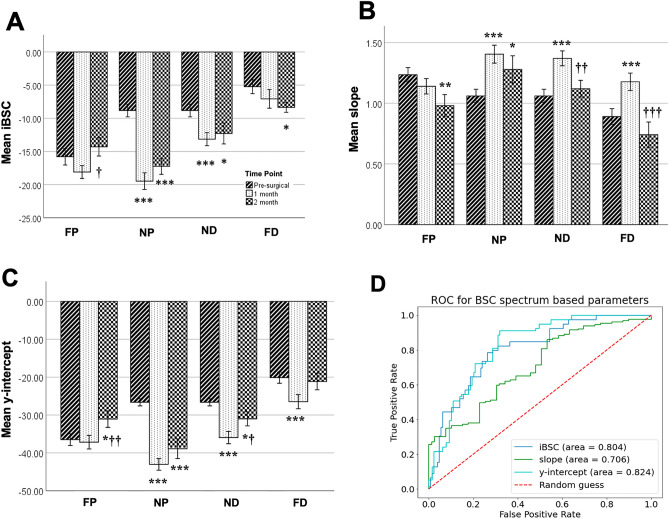
BSC-spectrum outcome at different time points and at different sites. The data was divided into four individual sites and one-way ANOVA across different time points was performed. Statistical results of **(A)** iBSC over selected bandwidth, **(B)** slope, **(C)** y-intercept linear fit were displayed through the bar plot. (*p < 0.05 vs pre-sur, **p < 0.01 vs pre-sur, ***p < 0.001 vs pre-sur, ^†^*p* < 0.05 vs 1 month, ^††^*p* < 0.01 vs 1 month, ^†††^*p* < 0.001 vs 1 month). mean ± SEM, **(D)** ROC curves (only included NP, ND at three-time points) for the three BSC spectrum parameters with AUC for iBSC = 0.81 (0.75, 0.87), y-intercept = 0.822 (0.77, 0.88), and slope = 0.30 (0.23, 0.38).

Attenuation compensated BSC outcomes of nerve degeneration were then evaluated across time points within each region; significant differences were found (Fig. 3 and Tables 1, 2, with individual BSC-frequency curves in Supplementary Figure S1a–c). The most substantial effects were observed in the NP and ND regions, with a significant main effect of time on iBSC, slope, and y-intercept. In both near regions, iBSC and y-intercept were significantly reduced, and slope significantly increased in 1-month post-surgery compared to pre-surgery (Fig. 3A–C). In the NP stump, changes in iBSC, slope, and y-intercept persisted through the two months of observation (Fig. 3A–C). In the FP region, despite increased cross-sectional area (Fig. 2), no main effect of time was observed on any BSC spectrum-based outcome, though post hoc testing revealed modest reductions in slope and corresponding increases in y-intercept in the FP region at the 2-month time point (Fig. 3B, C). In the FD region, the main effect of time was significant, with progressively reduced iBSC, transiently increased slope, and transiently reduced y-intercept for the first two months of degeneration. Similar significant decreases of iBSC in NP, ND, and FD regions were observed when iBSC was evaluated for a frequency range spanning the center frequency (18–26 MHz; Supplementary Figure S1d). Finally, ROC curves demonstrated good classifier performance of evaluation (area under the curve, AUC > 0.8) of the y-intercept and iBSC in distinguishing between pre-operative and injured nerves (Fig. 3D). However, slope only provided fair classifer performance as a diagnostic tool (AUC > 0.70).

**Table 1:** Post hoc test of *p* values across different imaging sites at each time point for different QUS-based parameters.

	Site a	Site b	iBSC *p* value	Slope *p* value	Y-intercept *p* value
Pre-surgery	FP	NP	< 0.001***	0.011*	< 0.001***
		ND	< 0.001***	0.011*	< 0.001***
		FD	< 0.001***	< 0.001***	< 0.001***
	NP	ND	NA	NA	NA
		FD	0.001**	0.014*	< 0.001***
	ND	FD	0.001**	0.014*	< 0.001***
1 month	FP	NP	0.254	0.002**	0.001**
		ND	< 0.001***	< 0.001***	0.487
		FD	< 0.001***	0.608	< 0.001***
	NP	ND	< 0.001***	0.633	< 0.001***
		FD	< 0.001***	0.002**	< 0.001***
	ND	FD	< 0.001***	0.007**	< 0.001***
2 month	FP	NP	0.034*	0.103	0.978
		ND	0.148	< 0.001***	< 0.001***
		FD	< 0.001***	0.005**	< 0.001***
	NP	ND	< 0.001***	0.063	< 0.001***
		FD	< 0.001***	< 0.001***	< 0.001***
	ND	FD	0.005**	< 0.001***	< 0.001***

FP: far proximal; NP: near proximal; ND: near distal; FD: far distal; iBSC: integrate backscatter coefficient. NA since at the pre-surgery time point, the same group was applied for NP and ND groups.

*p < 0.05; **p < 0.01;***p < 0.001.

**Table 2:** Post hoc test of *p* values across different imaging sites at each time point for different GLCM-based parameters.

	Site a	Site b	GLCM contrast *p* value	GLCM entropy value	GLCM correlation *p* value	GLCM energy *p* value	GLCM homogeneity *p* value
Pre-surgery	FP	NP	< 0.001***	0.011*	< 0.001***	0.011*	< 0.001***
		ND	< 0.001***	0.011*	< 0.001***	0.011*	< 0.001***
		FD	< 0.001***	< 0.001***	< 0.001***	< 0.001***	< 0.001***
	NP	ND	NA	NA	NA	NA	NA
		FD	< 0.001***	0.006**	< 0.001***	0.454	0.006**
	ND	FD	< 0.001***	0.006**	< 0.001***	0.454	0.006**
1 month	FP	NP	< 0.001***	< 0.001***	0.001**	< 0.001***	< 0.001***
		ND	< 0.001***	0.963	0.003**	0.379	0.805
		FD	< 0.001***	< 0.001***	< 0.001***	0.098	< 0.001***
	NP	ND	0.019*	< 0.001***	0.178	< 0.001***	< 0.001***
		FD	< 0.001***	< 0.001***	< 0.001***	< 0.001***	< 0.001***
	ND	FD	< 0.001***	< 0.001***	0.005**	0.01*	< 0.001***
2 month	FP	NP	0.063	0.015*	0.229	0.001**	0.009**
		ND	0.695	0.938	0.049*	0.414	0.781
		FD	< 0.001***	0.188	< 0.001***	0.897	< 0.001***
	NP	ND	0.143	0.019*	0.4431	0.017*	0.020*
		FD	< 0.001***	< 0.001***	< 0.001***	< 0.001***	< 0.001***
	ND	FD	< 0.001***	0.163	< 0.001***	0.344	< 0.001***

FP: far proximal; NP: near proximal; ND: near distal; FD: far distal. NA since at the pre-surgery time point, the same group was applied for NP and ND groups.

*p < 0.05;**p < 0.01;***p < 0.001.

**Figure 4:**
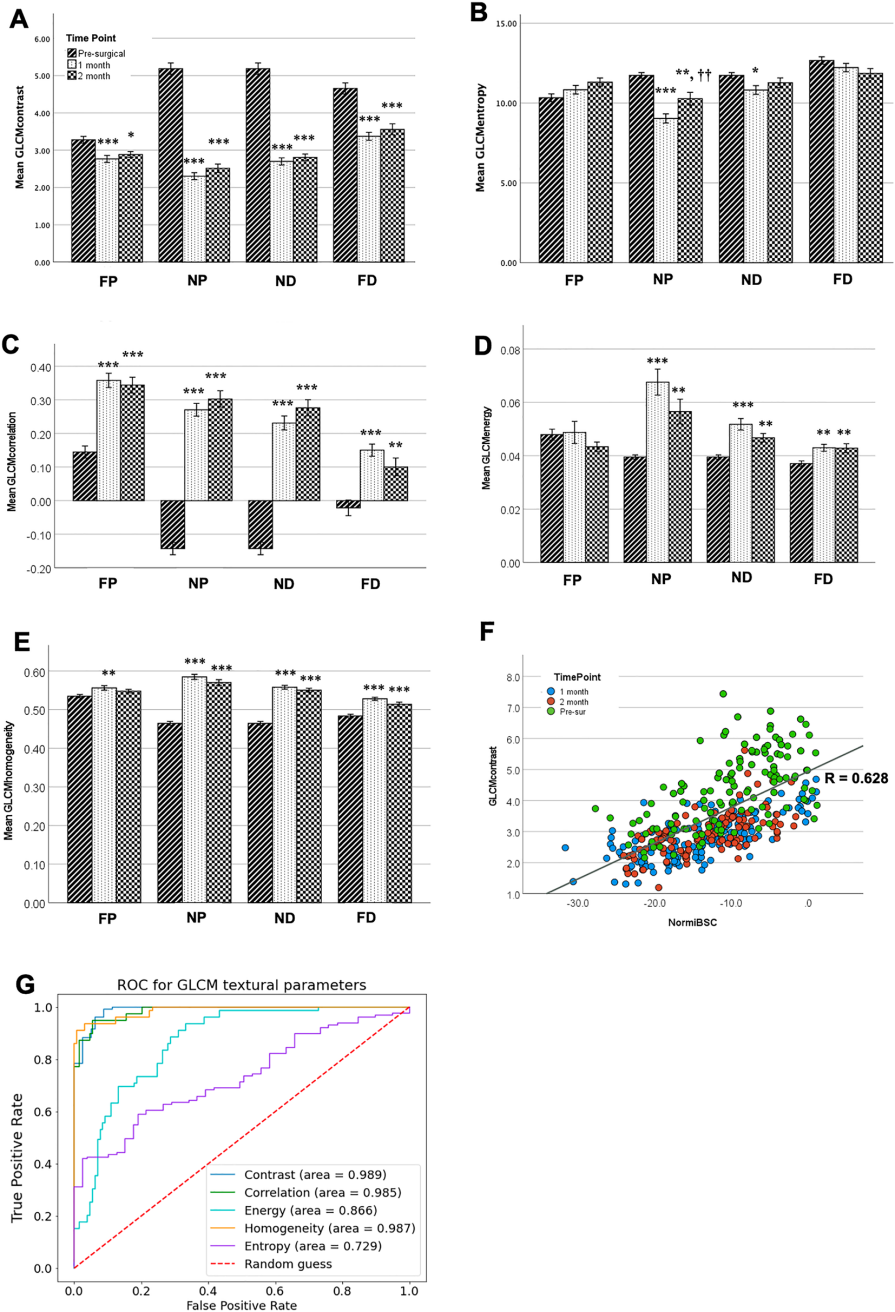
GLCM textural analysis of reconstructed B mode images from RF signal **(A–E)** the statistical outcome of GLCM contrast, Entropy, correlation, Energy, and Homogeneity at different time points and acquisition locations. (*p < 0.05, **p < 0.01, ***p < 0.001). mean ± SEM **(F)** scattering plot of iBSC versus GLCM contrast. Pearson correlation coefficient is 0.628 with p (two-tail) < 0.001. **(G)** ROC curves (only included NP, ND at three-time points) for the five GLCM parameters with AUC for contrast = 0.99 (0.98, 1.00), entropy = 0.73 (0.66, 0.79). (**H**) Correlation = 0.99 (0.98, 1.00), energy = 0.87 (0.81,0.91), and homogeneity = 0.99 (0.97,1.00).

We then performed GLCM-based analysis on B-mode images reconstructed from RFdata. Every GLCM parameter displayed significant changes in the NP region with reduced GLCM contrast and entropy, and increased GLCM correlation, homogeneity, and energy over the course of degeneration (Fig. 4A–E). In the ND region, only GLCM contrast, homogeneity, and correlation displayed a main effect of time. In the FP region, no main effect was observed for any GLCM parameter except for increased GLCM correlation. Finally, in the FD region, a main effect of time was observed for all GLCM parameters except entropy; reduced GLCM contrast and increased GLCM homogeneity, correlation, and energy were observed. ROC curves demonstrated excellent model discrimination of GLCM contrast, correlation, and homogeneity (Fig. 4G, AUC > 0.99). Good classifier performance of evaluation was observed for GLCM energy (Fig. 4G, AUC > 0.85), but only moderate classifier performance of evaluation was observed for GLCM entropy (Fig. 4G, AUC > 0.70) in classifying pre-operative and post-operative data.

We further investigated the relationship between BSC spectrum-based measurements and GLCM -based measurements using regression analysis (Fig. 4F and Table 3). GLCM contrast was significantly correlated with iBSC (r = 0.621, *p* < 0.001). Though all experimental groups were well represented across the regressed domains, pre-op values of GLCM contrast and iBSC were generally located in the upper right domain of the regression plot (green symbols), with post-op values more biased to the lower left (red and blue symbols); these values are consistent with mean shifts observed in Figs. 3A and 4A. The correlation matrix among all imaging parameters is summarized in Table 3. Briefly, GLCM contrast and GLCM entropy positively correlated with iBSC and y-intercept; GLCM correlation, homogeneity, and energy negatively correlated with iBSC and y-intercept; and slope had a negative correlation with GLCM parameters.

**Table 3:** Pearson correlation coefficient matrix among GLCM, BSC spectrum-based, and area with correlation at the 0.01 significance level (2-tailed).

Variables	iBSC	Slope	Y-intercept	GLCM Contrast	GLCM Correlation	GLCM Homogeneity	GLCM Energy	GLCM Entropy	Area
iBSC	1								
Slope	− 0.284	1							
Y-intercept	0.842	− 0.753	1						
GLCM Contrast	− 0.628	− 0.418	0.661	1					
GLCM Correlation	− 0.462	0.243	− 0.446	− 0.818	1				
GLCM Homogeneity	− 0.641	0.447	− 0.689	− 0.953	0.761	1			
GLCM Energy	− 0.529	0.429	− 0.607	− 0.562	0.229	0.711	1		
GLCM Entropy	0.695	− 0.434	0.726	0.580	− 0.204	− 0.640	− 0.755	1	
Area	− 0.279	0.243	− 0.327	− 0.630	0.628	0.612	0.224	− 0.101	1

**Figure 5:**
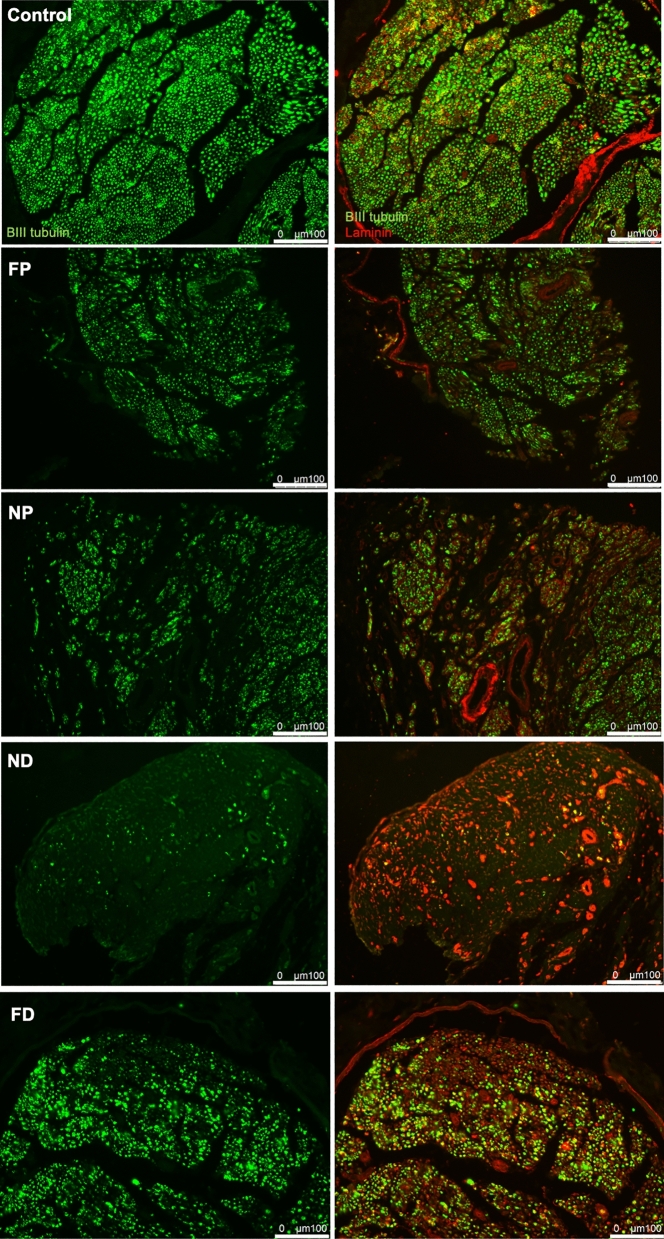
Immunohistochemistry (IHC) outcome at 1-month post-injury. Each row represents a different location to the nerve. The first column is the BIII tubulin and the second column is overlay images. Green is Beta III tubulin. Red is Laminin.

**Figure 6:**
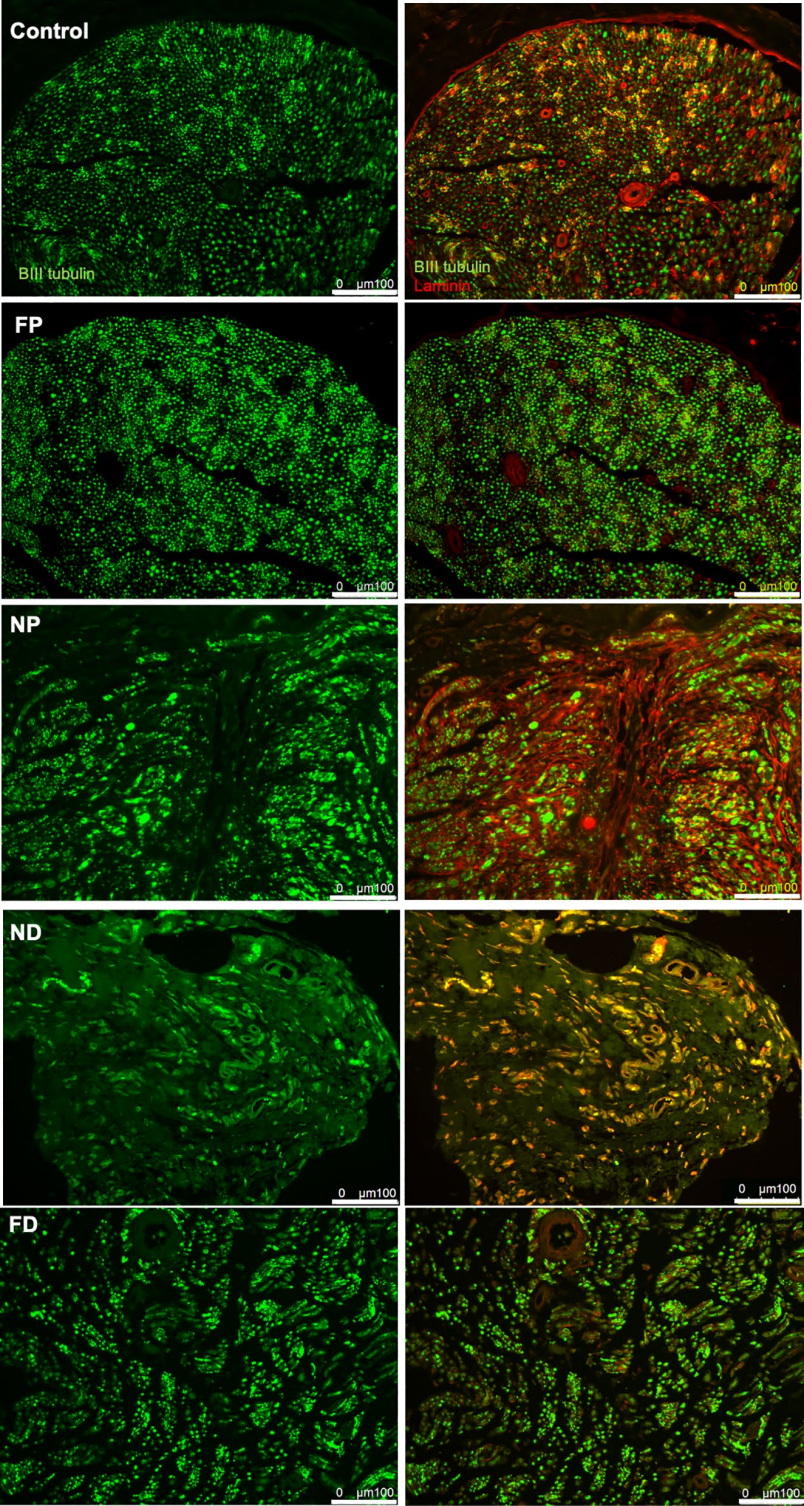
Immunohistochemistry (IHC) outcome at 2-month post-injury. Each row represents a different location to the nerve. The first column is the BIII tubulin and the second column is overlay images Green is Beta III tubulin. Red is Laminin. The raw images’ contrast were adjusted for visualization purposes.

Finally, to test whether US-based outcomes reflected regional and temporal morphological changes expected with nerve injury without repair^51–53^, IHC outcomes of injured ipsilateral and contralateral control nerves were inspected at each time point (Figs. 5, 6, Supplementary Figure S2a). Contralateral sciatic nerves and far proximal stumps retained normal axon structure and organization, characterized by punctate axons surrounded by characteristic laminar rings. Regional heterogeneity as a response to injury was observed, with axon and laminin organization better retained in FP locations than in NP and ND locations at all time points. Increased distal axonal loss and disorganization of axon-positive labeling were observed at 1- and 2-month post-op time points (Figs. 5, 6, Supplementary Figure S2a). The ND region especially showed the loss of the structural integrity of both axons and basal lamina.

**Figure 7:**
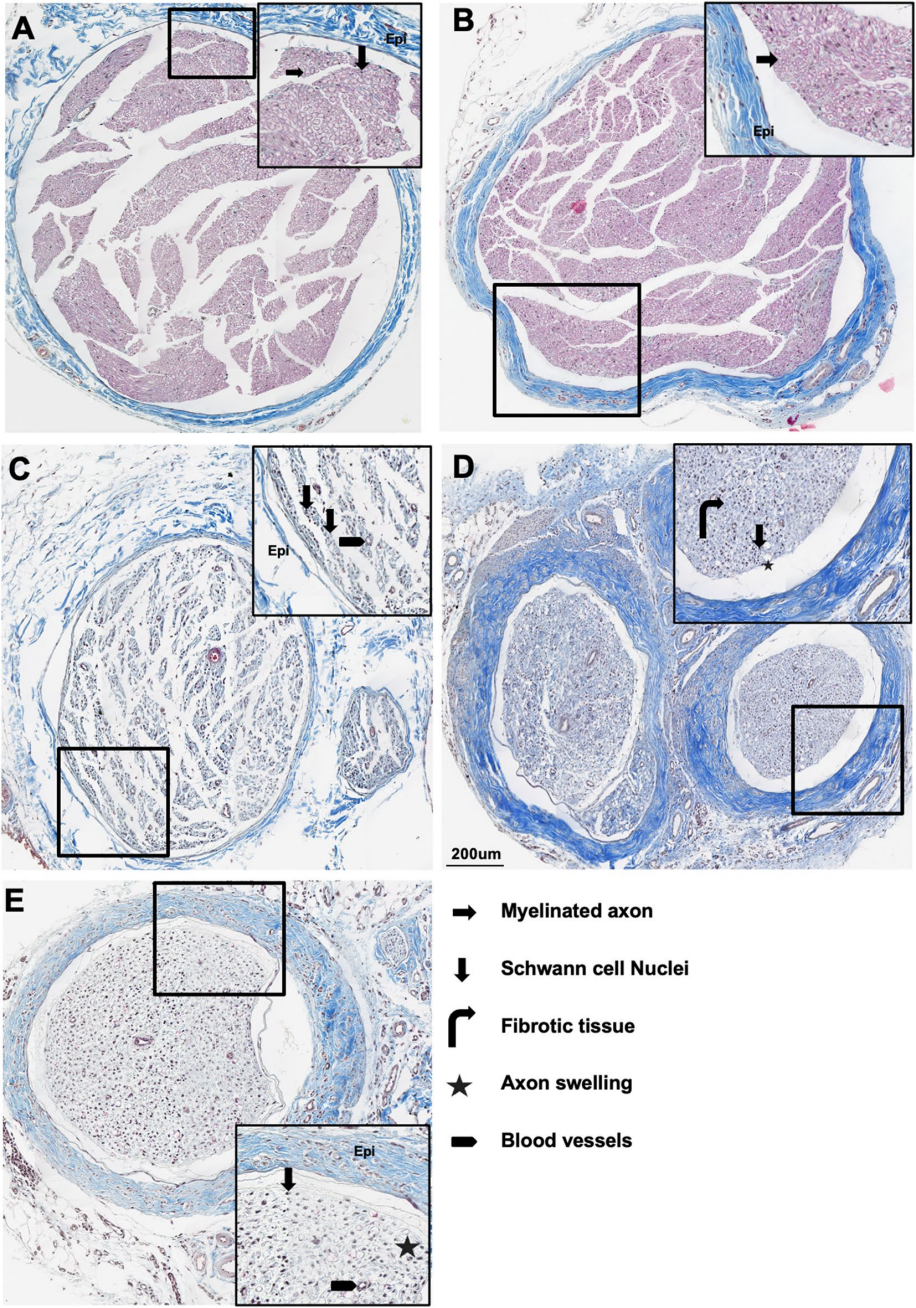
Trichrome outcome at 1-month post-injury. **(A)** Contralateral nerve, **(B)** FP, **(C)** NP, **(D)** ND, **(E)** FD. Axon (right arrow). Schwann cell nuclei (Down arrow). Axon degeneration, axon swelling (star).

**Figure 8:**
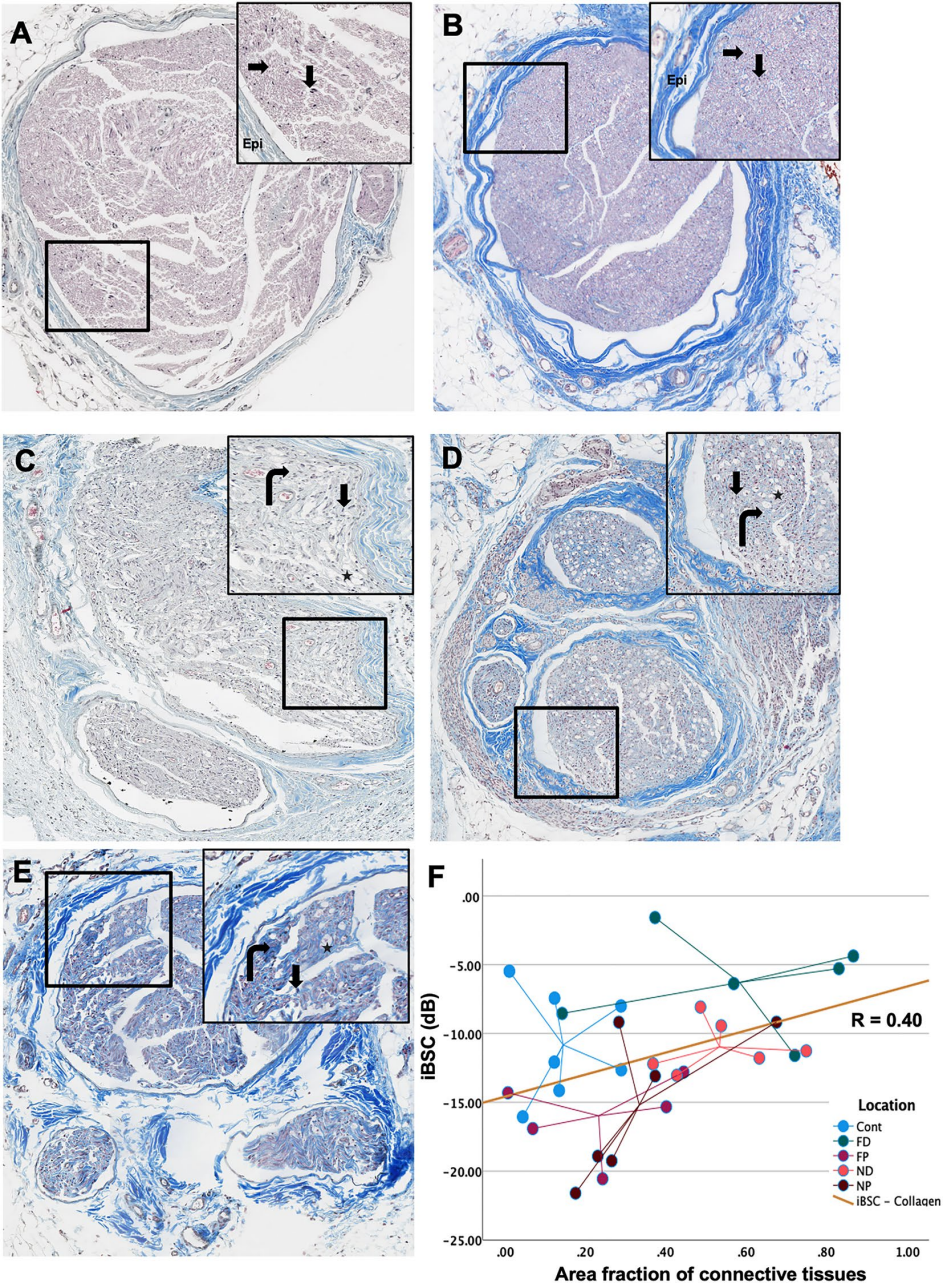
Trichrome outcome at 2-month post-injury. **(A)** Contralateral nerve, **(B)** FP, **(C)** NP, **(D)** ND, **(E)** FD. Axon (right arrow). Schwann cell nuclei (Down arrow). Axon degeneration, axon swelling (star). **(F)** The scatter plot between iBSC and area fraction of connective tissues quantified by trichrome staining images with different location labeled. The Pearson correlation relationship is R = 0.40, *p* (two-tail) = 0.028.

The Masson Trichrome outcomes of injured and contralateral nerves were also examined regionally and temporally, Figs. 7, 8). Contralateral sciatic and FP stumps exhibited normal axon structure and organization with healthy myelin sheath around the axon. The blue epineurium is intact and relatively thin surrounding the fascicles (Figs. 7, 8A, B). The chronic injury-induced heterogeneity and disorganization in close injury location at both time point demonstrate following structural changes. The epineurium post-injury thickened; schwann cell myelin sheath disappeared (less red component); more fibrotic tissues infiltrate inside the nerve fascicles (more blue component). Other than the disorganization and fibrotic tissue infiltration, more small blood vessels and axonal swelling were also observed. As a first step towards understanding a structural basis for BSC-based parameters, regression analysis was performed. Though there was no correlation between iBSC and axon density (r = -0.08, p = 0.73), a significant (albeit modest) positive correlation was observed between the iBSC and collagen ratio (r = 0.4, p = 0.028) Supplementary Figure S2b, Fig. 8F).

## Discussion

This study investigated two questions: (1) are BSC spectrum-based and GLCM-based parameters calculated from high-frequency US capable of revealing degenerative changes in nerve structure following unrepaired nerve transection injury? (2) Given that these outcomes reflect different, albeit potentially interlinked, approaches to quantifying tissue response to acoustic waves, is there a correlation between the BSC spectrum-based and GLCM-based parameters?

In order to answer these questions, we tested the efficacy of non-invasive, quantitative high-frequency US in a rat model of chronic peripheral nerve injury. The results demonstrated that both BSC spectrum-based and GLCM-based parameters detect progressive differences in sciatic nerve structure following peripheral nerve injury (Figs. 3, 4), revealing spatial and temporal differences beyond those suggested by geometric assessments of area of ROI alone (Fig. 2). Moreover, many BSC spectrum-based parameters strongly correlated with GLCM-based parameters (Fig. 4F and Table 3), providing evidence that there might be intrinsic biological sources that influence both sets of parameters. These results demonstrate the potential for QUS and GLCM analysis as non-invasive diagnostic tools for evaluating nerves after injury.

### Nerve degeneration model

A number of observations validated our rat sciatic nerve injury model, providing confidence that BSC spectrum-based and GLCM-based outcomes reflected the consequences of nerve injury and degeneration. Our use of small PDMS barriers to inhibit spontaneous regrowth was analogous to capping schemes in other chronic injury models^54^. Serendipitously, these blocks served as imaging reference markers due to their hypo-echogenicity^55^, providing a spatial reference point when assessing regional properties. Progressive regional changes in nerve morphology, especially in regions close to the injury site and the distal stump^52^, as well as muscular atrophy^56^, were consistent with nerve degeneration. B-mode images and immunohistochemistry reflected these outcomes. The cross-sectional images of the sciatic nerve at the pre-injury time point showed a consistent size and a thin hyper-echoic epineurium. Nerve response to injury without repair was generally as expected based on prior literature on nerve degeneration^52, 57^. At post-injury time points, nerve cross-sections in the far proximal region, which contain axons still physically and biologically connected to cell bodies in the spinal cord but are some distance from the injury site, had similar epineural and intra-epineural echogenicity as for the pre-injury time point, despite increased cross-sectional area (Fig. 2). However, closer to the injury site, both NP and ND nerve locations acquired a disorganized epineurium structure, thicker nerve border, and more homogeneous and hypoechogenic inner nerve post-injury compared with pre-injury^58, 59^. The shapes of the nerve stumps also changed significantly post-injury, both due to injury and surgical manipulation and decompression of the nerve. For example, at the NP stump, the nerve dilated from the pre-surgery time point by ~ 3x, presumably due to edema and/or neuroma formation. At the FD location, a thicker epineurium and reduced echogenicity of the inner nerve structure were observed, consistent with Wallerian degeneration^3, 5^.

### Changes in BSC spectrum-based and GLCM-based parameters after nerve injury

Pre-injury US outcomes varied along the length of the sciatic nerve, consistent with typical changes in nerve geometry, internal fasciculation, and external branching. While B-mode images revealed qualitative changes in echogenicity and apparent changes in size among groups, such measures are susceptible to being confounded by differences in system or user. Thus, quantitative measures were employed, and successfully distinguished between injured and non-injured nerves, with iBSC and y-intercept showing high sensitivity (Fig. 3). Significant regional changes in all three BSC spectrum-based parameters (iBSC, slope, and y-intercept) were observed at post-injury time points (Figs. 3, 4). Despite the small size of the imaged and analyzed ROI, a similar order of magnitude of iBSC (~ − 10 dB) was measured in human nerves^30, 31^. As expected based on distance from injury site and retained connectivity to neuronal cell bodies, BSC spectrum-based parameters in the FP regions of the nerve did not change appreciably with time (Figs. 3, 4), indicating relatively healthy tissue structure even with the chronic nerve injury (Figs. 5, 6, 7B and 8B). In contrast, the NP and ND locations displayed significant changes in BSC spectrum-related parameters (Fig. 3), despite the persistence of axons—albeit disorganized—approaching the proximal face of the PDMS block. Finally, at the FD location, significant differences were noticed pre- and post-surgery, with iBSC and y-intercept fluctuating over time. These outcomes reflect the complexity of the FD region. On one hand, the degeneration continues without neural input leading to the infiltration of fibrotic tissues, epineurium thickening and increasing small blood vessels (Figs. 7, 8); on the other hand, consistent with outcomes in this and prior studies, the FD stump maintains a level of structural integrity greater than sites closer to the site of injury^52, 60^ (Figs. 5, 6).

Reductions in iBSC are consistent with reduction in scattering intensity as nerve injury progresses, as also implied by reduced intra-epineurial echogenicity in B-mode images. Beyond echogenicity, though, these outcomes also suggest additional insight into structural remodeling that cannot be gleaned from simple geometric and macroscopic echogenicity measurements. The underlying influences on the BSC-spectrum based outcomes are complex and a source of current discussion. Early work demonstrated that BSCs could be described by two independent parameters, suggesting a linear model fit for BSC-frequency relationships in the liver^39^. They also suggested that some characteristics of the scatterers within the tissue, including their effective size, concentrations/densities, and relative acoustic impedance, may be connected to BSC descriptors^61, 62^. More recently, Muleyki-Seya and colleagues^63^ investigated a Gaussian model applied to data measured in mouse liver, with the intent of matching two model parameters—Effective Scatterer Diameter (ESD) and Effective Acoustic Concentration (EAC)—with empirical parameters proposed by Lizzi-Feleppa. Though these approaches are reasonable for relatively homogenous tissues, nerves do not meet key assumptions underlying such models (e.g., Gaussian scatterers randomly distributed in a dilute medium). Thus, though we demonstrate a significant correlation between connective tissue density and iBSC (Fig. 8), we cannot provide a full description of scattering elements in nerve.

While such evaluation is well beyond the scope of this study, we posit that collagen fibers in the epineurium and perineurium as well as individual nerve fibers, including axons and their surrounding basal lamina and endoneurial connective tissue, are the most likely scattering elements, based on prior data from our group detailing a strong correlation between iBSC and collagen and myelin levels in cadaveric nerves^31^. However, future studies are required to test these possibilities, which integrate quantitative IHC data, ultrasound-based outcomes, and more sophisticated theoretical analysis (e.g., polydisperse models^64^). One possibility may be to compare different scattering models, underlying assumptions and comparative validity among which may provide clues as to the identity of key scattering elements. Alternatively, IHC outcomes could underpin a solution to the inverse problem, providing a theoretical BSC that may be compared to experimental BSC to gain insight into scatterer identity.

GLCM-based texture analysis also exhibited strong diagnostic accuracy/sensitivity to chronic injuries (Fig. 4G). GLCM contrast and entropy were significantly reduced after the injury, and GLCM homogeneity, energy, and correlation significantly increased at NP and ND locations (Fig. 4A–F), These observations are consistent with a more homogeneous speckle-pattern in B-mode images since the relative contrast between neighboring pixels, per the GLCM algorithm, is smaller. Similar observations were also noted in previous textural analysis of peripheral nerves, in which diminished GLCM contrast was observed with the progression of amyotrophic lateral sclerosis^65^. Biologically, decreased contrast and entropy could be explained by reduced laminin integrity, lower axonal density, and altered epineurial integrity^66^ (Figs. 5, 6). In this study, GLCM-based parameters appeared to be more sensitive than the BSC spectrum-based measurements (Figs. 4G vs. 3D). This may be a consequence of the scale of correlative analysis of this set of methods relative to the ROI compared to attenuation and BSC-based methods, which may be more robust for larger ROIs. This may also be a consequence of underlying assumptions of medium homogeneity underlying conventional attenuation-based analysis, even after accounting for overlying tissue, and high sensitivity of attenuation-based outcomes to shadowing^30, 67^. GLCM-based analysis is highly dependent on a given system’s operator-dependent settings, such as time gain compensation, depth of focus, and post-processing digital/analog filters^68^. Application and interpretation of textural outcomes should weigh such criteria.

Given the potential differences in fundamental bases for BSC- and GLCM-based outcomes (e.g., bulk properties vs. local variation in acoustic response), correlations between BSC-based and GLCM-based parameters were also investigated (Fig. 4F and Table 3). Numerous strong positive and negative correlations were observed among multiple pairs of parameters. Among these, a positive correlation between iBSC and GLCM contrast was observed, which is consistent with previous literature in cadaveric human ulnar nerves—a tissue varying in both composition and scale^31^. We speculate that for BSC spectrum-based parameters, the loss of axons or their laminar/connective tissue sheaths contributes to the loss of effective scattering elements. For the GLCM parameters, degenerative processes reduce the hypoechoic appearance of the interior of nerve fascicles compared with the hyperechoic surrounding structures—i.e., diminished interfascicular connectivity^58, 69^. Based on these principles, decreased iBSC and y-intercept measurements may positively correlate with decreased GLCM contrast and entropy^65, 70^.

### Limitations

In this study, we utilized literature-based values to compensate for the attenuation of overlying tissue. This approach improved the stability of BSC spectrum-based measurements by avoiding artifactual effects of layered interfaces^30^. However, possibly meaningful biological impacts of overlying tissues on acoustic response are ignored. In addition, the attenuation of nerve itself was not included in the compensation, due to its small size and lack of literature on attenuation values at high frequency. An alternative approach to reducing the impacts of tissue layers and heterogeneity is the application of analytical methods that are less dependent on tissue homogeneity, such as the spectral log difference method or regularized spectral log difference method^71, 72^. Such approaches may overcome the limitations of methods such as the spectral difference method, which assume the homogeneity of an image medium^73, 74^. Phantoms that better mimic the nerve, which may be considered a multilayered and transversely isotropic tissue, may also drive improvements in analytical approaches. For example, a previous study demonstrated the efficacy of layered phantoms with different attenuation coefficients^75^. More sophisticated nerve-mimicking phantom should be built in the future for improved validation of QUS outcomes.

Although PDMS is a relatively biologically inert material^76^, as with all implants, there is a possibility of inflammation and granulation of tissue^77^. Such phenomena could have a disproportionate effect on measurements at ND and NP stumps. For instance, iBSC could be higher since components of granulation tissue could serve as more highly scattering and GLCM contrast could be lower since these tissues are as hyperechoic to the surrounding tissues^78^. However, the PDMS block was fabricated to minimize its footprint, implants were retrieved cleanly after animal sacrifice, and measurements were made in regions where the epineurium appeared intact (even if thickened), suggesting that such impacts were unlikely to be substantial. Furthermore, peak inflammatory responses happen around 14 days post-surgery^3, 5^, so our evaluation at a 1-month time point is after these dynamics have subsided. On the other hand, minimizing PDMS block size may reduce the efficiency of inhibiting axonal regeneration. While axon-positive labeling was sparse in distal stumps, we cannot rule out minor contributions of spontaneously regenerating axons to ultrasound signatures.

From an experimental design standpoint, sample sizes are imbalanced across time points, due to the sacrifice of a subset of animals at each time point for histological analysis. While this allowed larger sample sizes at earlier time points and the potential for longitudinal analysis in some animals (a fact that was ultimately not important), in the future, equalizing sample sizes may provide statistical simplicity, and allow a balanced correlation between US and IHC outcomes for a full data set. Finally, we elected to use only young male rats in this study, to represent the young male demographic that is most susceptible to nerve injury^79, 80^. However, based on age- and gender-related differences in nerve regeneration^81, 82^, future studies should include such factors.

## Conclusion

This study investigated the efficacy of QUS BSC spectrum-based and GLCM-based techniques in evaluating chronic peripheral nerve injury in vivo. Both strategies have the potential to be translated clinically for diagnosing nerve injury. BSC spectrum-based parameters have considerable power based on their system independence, but require the availability of a reference phantom appropriate for the deployed scanning frequency. However, if RF data acquisition is unavailable clinically or is not usable due to high tissue heterogeneity, GLCM-based analysis may be an alternative diagnostic tool; care must be taken to standardize acquisition settings for such B-mode assessments. In conclusion, this work has important implications for understanding the application of quantitative high-frequency US approaches to the evaluation of peripheral nerve injury and disease.

### Supplementary Information

Supplementary Information.

## Data Availability

The final datasets from the current study will be made available upon written request to corresponding author (SBS) in accordance with the Open Data policy mandated by the Department of Veterans Affairs.
