# Supplementary material for: Quantitative evaluation of rat sciatic nerve degeneration using high-frequency ultrasound
Source: Sci Rep. 2023 Nov 18;13:20228. doi: 10.1038/s41598-023-47264-9 (PMC10657462; doi:10.1038/s41598-023-47264-9)
Supplement: Supplementary file 1 — Supplementary Information. [file 41598_2023_47264_MOESM1_ESM.pdf]

Quantitative evaluation of rat sciatic nerve degeneration using high-frequency ultrasound

Yuanshan Wu[1,3], Victor Barrere[2,3], Aiguo Han[4], Michael P. Andre[3,5], Elisabeth Orozco[2,3], Xin Cheng[3,5], Eric Y. Chang[3,5], Sameer B. Shah[1,2,3]*

[1]Department of Bioengineering, University of California, San Diego, United States

[2]Department of Orthopaedic Surgery, University of California, San Diego, United States

[3]Research Service, VA San Diego Healthcare System, San Diego, United States

[4]Department of Biomedical Engineering and Mechanics, Virginia Polytechnic Institute and State University, Blacksburg, VA, United States

[5]Department of Radiology, University of California, San Diego, United States

* Address correspondence to:

9500 Gilman Drive, MC 0863

La Jolla, CA 92093-0683

Email: sbshah@health.ucsd.edu

Phone: 1-858-822-0720

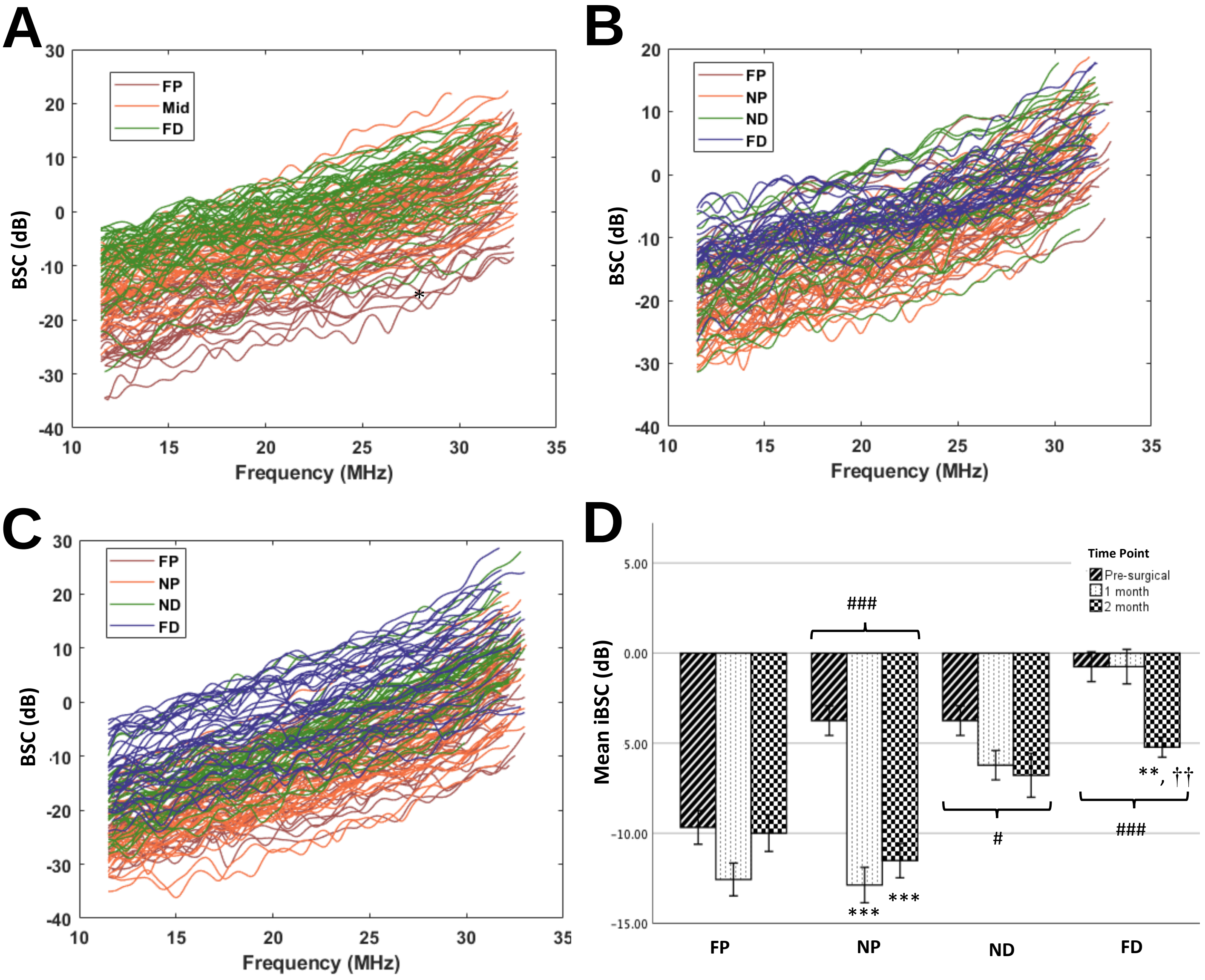

Supplementary Figure S1: Raw mean BSC curves and mean BSC curves at **a)** presurgical, **b)** 1-month, and **c)** 2-month post surgical (mean ± s.e.m). **d)** iBSC measurements from a different analysis bandwidth (18-26 MHz). (# $p < 0.05$, ### $p < 0.001$ with one-way ANOVA test, * $p < 0.05$ vs pre-sur, ** $p < 0.01$ vs pre-sur,*** $p < 0.001$ vs pre-sur, † † $p < 0.01$ vs 1 month, † † † $p < 0.001$ vs 1 month,). mean ± s.e.m

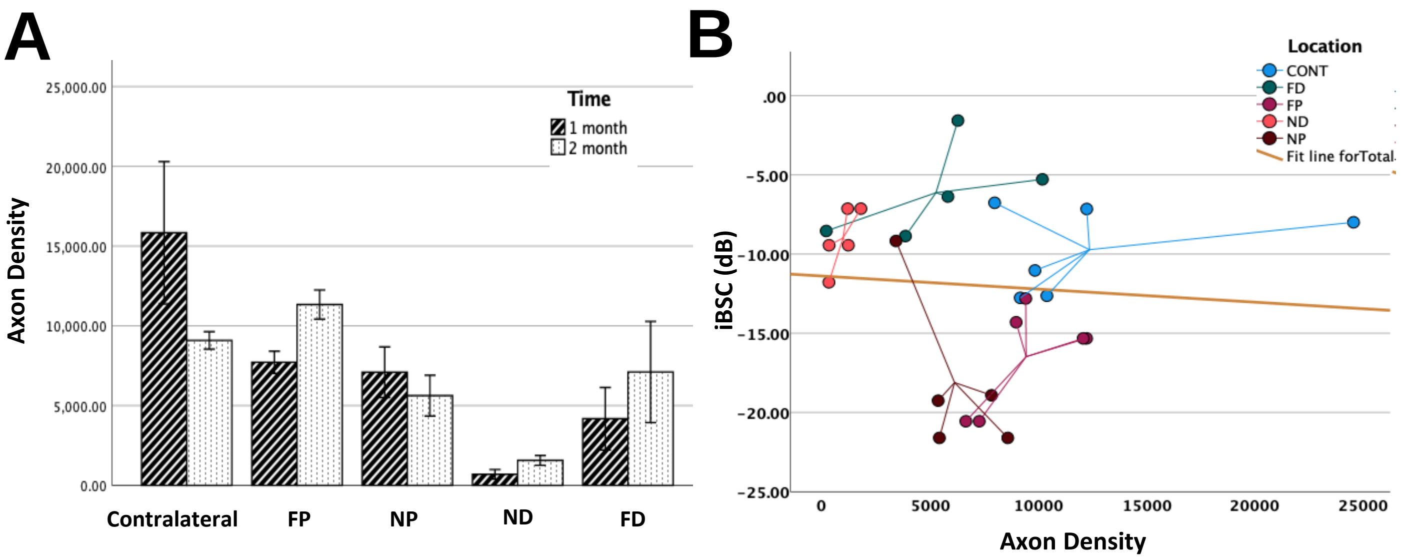

Supplementary Figure S2. **a)** The axon density quantified from IHC outcomes from different location. Note: most of the histological slices were taken from proximal side of contralateral leg. **b)** Scatter plot of iBSC measurements vs. IHC axon density measurements. No statistically significant correlation was observed.
